# Supplementary material for: Ubiquitination of GRK2 Is Required for the β-Arrestin-Biased Signaling Pathway of Dopamine D2 Receptors to Activate ERK Kinases
Source: Int J Mol Sci. 2023 Jun 12;24(12):10031. doi: 10.3390/ijms241210031 (PMC10298151; doi:10.3390/ijms241210031)
Supplement: Supplementary file 1 [file ijms-24-10031-s001.zip › ijms-2387203-supplementary.pdf]

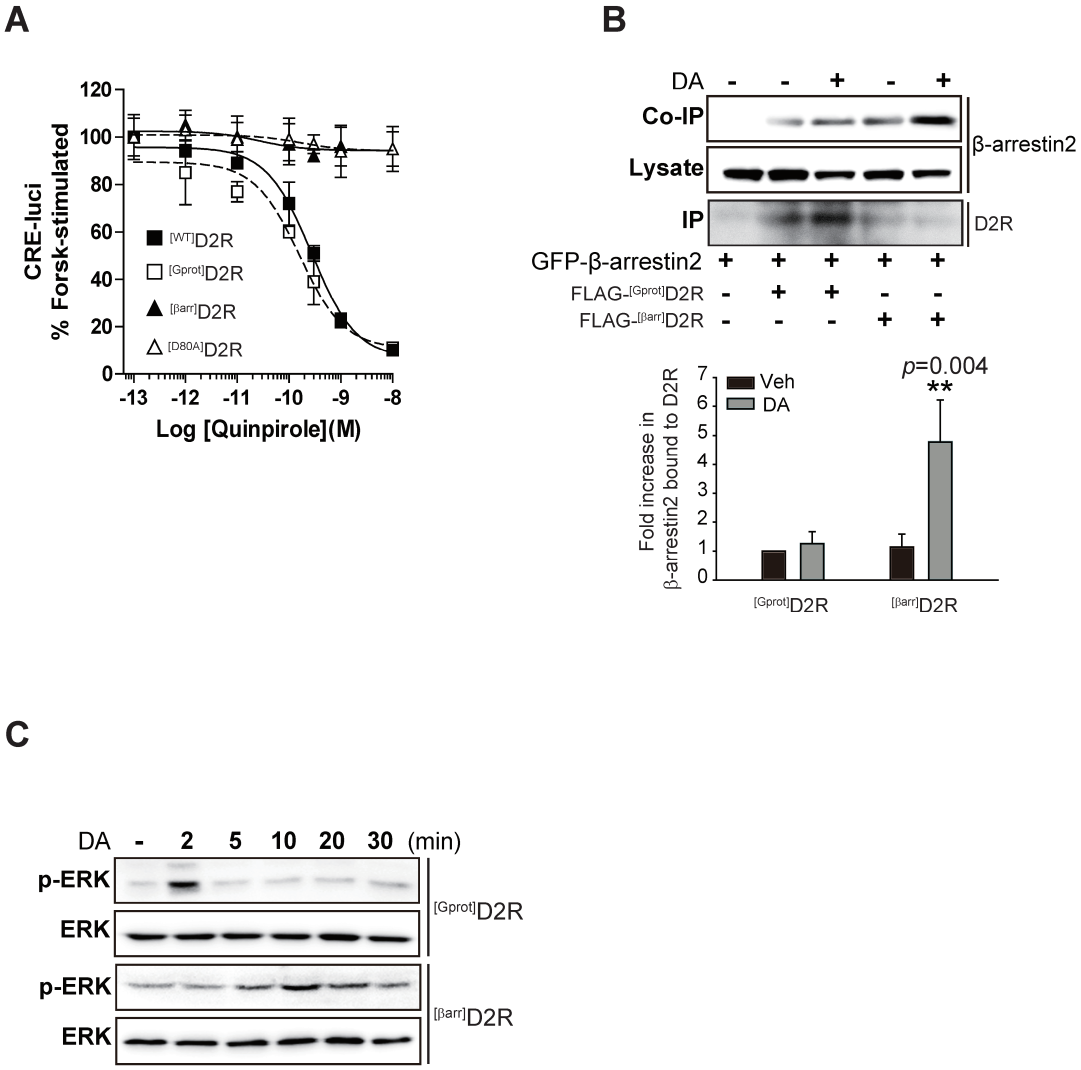

Figure S1. Characterization of [Gprot]D2R and [barr]D2R. (A) HEK 293 cells were transfected with plasmids encoding [WT]D2R, [Gprot]D2R, [βarr]D2R or [D80A]D2R. The cells were treated with increasing concentrations of quinpirole. The cellular levels of cAMP were evaluated by using the CRE-luciferase reporter gene assay. "Forsk" represents forskolin. (B) HEK 293 cells were transfected with plasmids encoding GFP-β-arrestin2 and FLAG-[Gprot]D2R or FLAG-[βarr]D2R. The cells were treated with either vehicle or 10 μM DA for 5 min. Cell lysates were immunoprecipitated using FLAG beads. Co-IP/Lysate and IP were immunoblotted with antibodies against GFP (1:1000 dilution) and FLAG (1:1000 dilution), respectively. **$p < 0.01$ compared with Veh/[βarr]D2R group (n=3). (C) HEK 293 cells were transfected with plasmid encoding either [Gprot]D2R or [βarr]D2R. Following serum-starvation, the cells were exposed to 10 μM DA for the indicated time points (0-30 min). Cell extracts were prepared as described in the "Materials and Methods" section and separated on 10% polyacrylamide gels. The levels of activated ERK1/2 were determined using a monoclonal antibody specific to the phosphorylated form of ERK1/2 (p-ERK1/2).

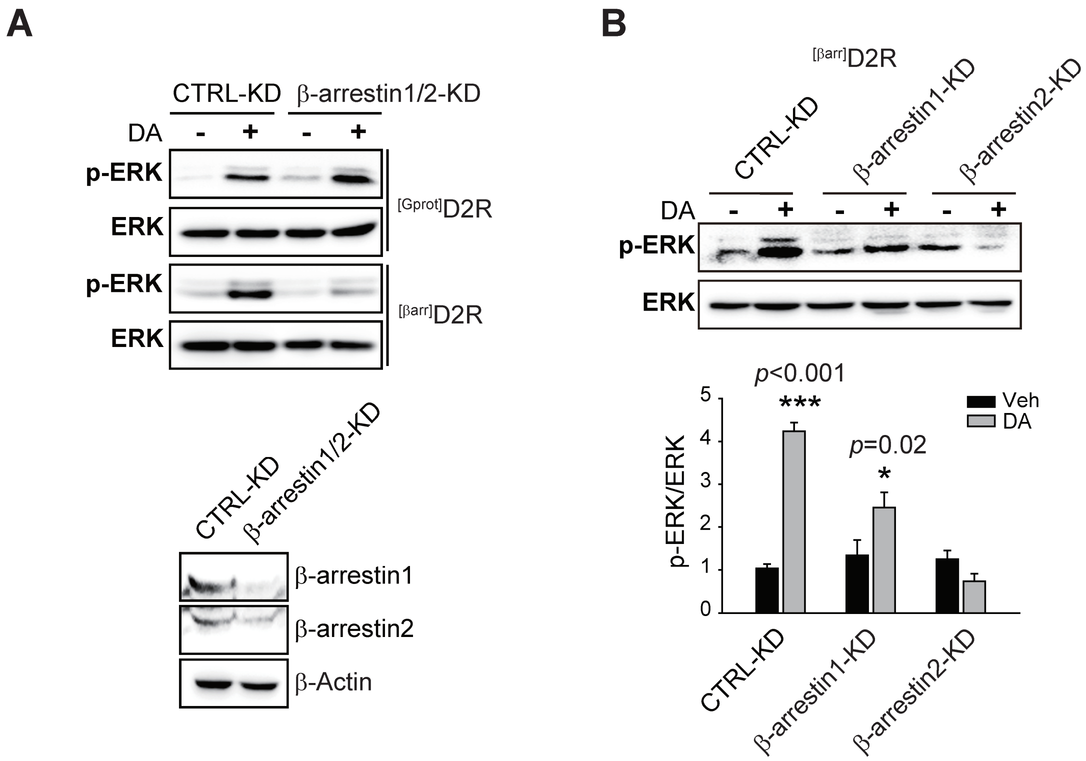

Figure S2. β-arrestin2 is involved in D2R β-arrestin pathway-mediated ERKs activation. (A) [Gprot]D2R or [βarr]D2R plasmid was transfected into CTRL-KD and β-arrestin1/2-KD cells. The cells were then treated with 10 μM DA for 2 min ([Gprot]D2R producing) or 10 min ([βarr]D2R producing). Immunoblotting with antibodies against p-ERK1/2 and ERK2 was performed on the lysates. The lysates of CTRL-KD and β-arrestin1/2-KD cells were immunoblotted with antibodies against β-arrestins (1:1000 dilution) and actin (1:2000 dilution), resulting in a reduction of cellular β-arrestin1 or β-arrestin2 levels by approximately 90% or 71%. (B) CTRL-KD, β-arrestin1-KD and β-arrestin2-KD cells were transfected with [βarr]D2R and treated with 10 μM DA for 10 min. Immunoblotting with antibodies against p-ERK1/2 and ERK2 was performed on the lysates. The data presented are the results of three independent experiments with similar outcomes. Statistical analysis showed significant differences with ***$p < 0.001$ and *$p < 0.05$ compared to the corresponding Veh group (n=3).

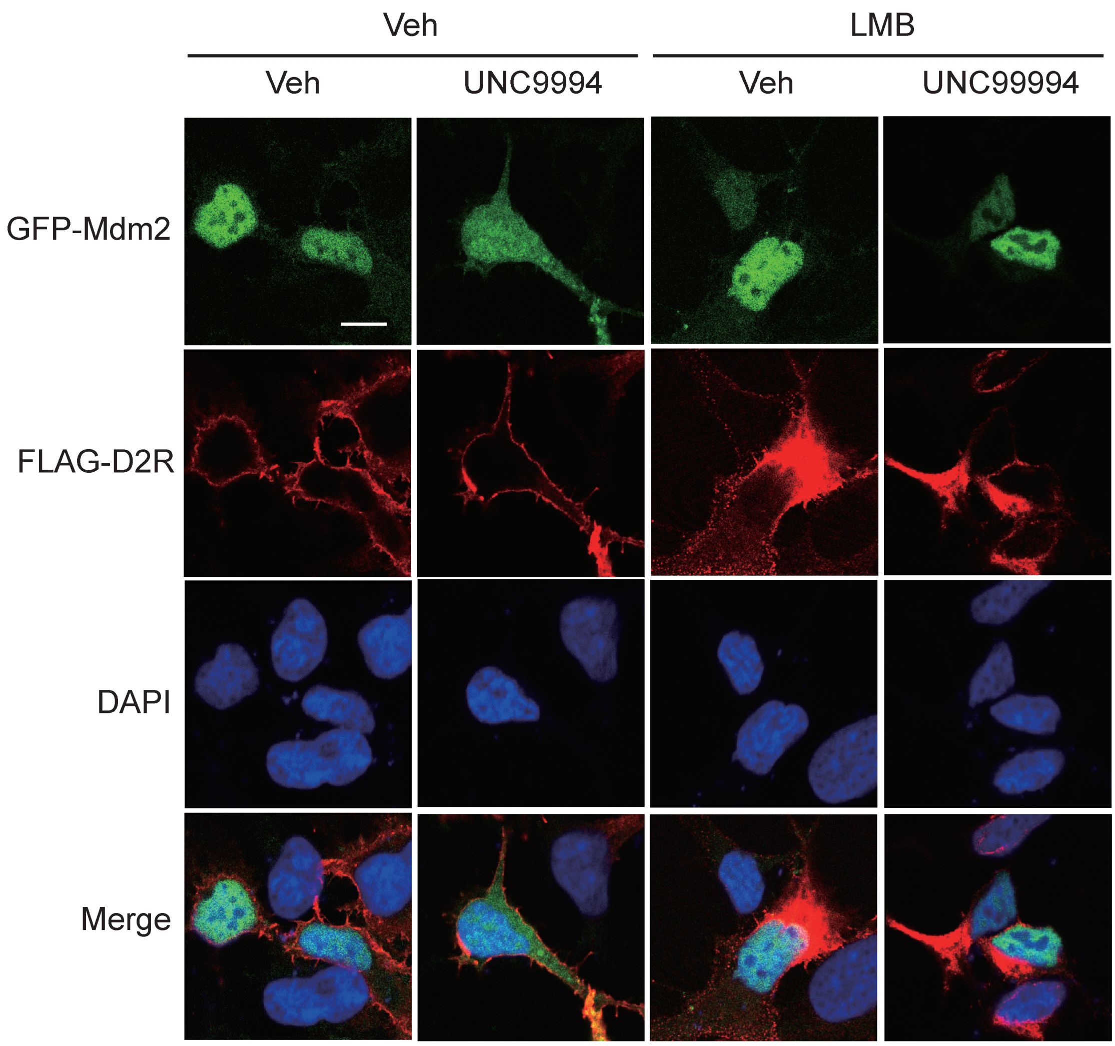

Figure S3. Pretreatment of LMB blocks UNC9994-mediated nuclear export of Mdm2. HEK 293 cells were transfected with plasmids encoding FLAG-D2R and GFP-Mdm2. After 24-36 h, cells were pretreated with either vehicle or 10 ng/ml LMB for 3 h, followed by treatment with 1 μM UNC9994 for 2 min. Then the cells were labeled with FLAG antibodies (1:1000 dilution), and then with Alexa 594-conjugated anti-rabbit secondary antibodies (1:500 dilution). The horizontal bar represents 10 μm. Data shows one representative example out of three independent experiments.

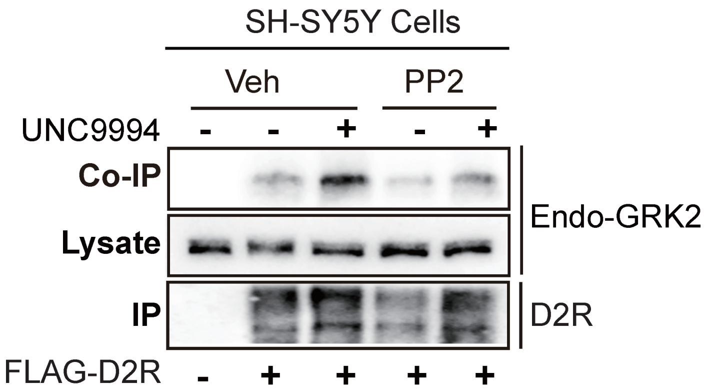

Figure S4. PP2 pretreatment blocked the interaction of Endo-GRK2 with D2R upon stimulation with UNC9994 in SH-SY5Y cells. HEK 293 cells were transfected with plasmids encoding D2R. Cells were pretreated with 10 μM PP2 for 30 min, followed by 1 μM UNC9994 treatment for 2 min. Immunoprecipitation of cell lysates using FLAG beads. Antibodies against GRK2 (1:2000 dilution) and FLAG (1:1000 dilution) were used to immunoblot Co-IP/Lysate and IP, respectively.
